# Diversity, distribution, and sustainability of traditional medicinal plants in Kaski district, western Nepal

**DOI:** 10.3389/fphar.2022.1076351

**Published:** 2022-12-20

**Authors:** Dhruba Khakurel, Yadav Uprety, Gyeongik Ahn, Joon-Yung Cha, Woe-Yeon Kim, Sung-Ho Lee, Sangeeta Rajbhandary

**Affiliations:** ^1^ Department of Biology, Graduate School, Gyeongsang National University, Jinju, South Korea; ^2^ Central Department of Botany, Tribhuvan University, Kirtipur, Nepal; ^3^ Research Institute of Life Science, Institute of Agriculture and Life Sciences, Gyeongsang National University, Jinju, South Korea; ^4^ Division of Applied Life Science (BK21four), Plant Molecular Biology and Biotechnology Research Center, Graduate School of Gyeongsang National University, Jinju, South Korea; ^5^ Division of Life Science, Gyeongsang National University, Jinju, South Korea

**Keywords:** ethnobotany, Gurung ethnic group, medicinal plants, overharvesting, traditional knowledge

## Abstract

Medicinal plants are the primary source of traditional healthcare systems in many rural areas mostly in developing countries. This study aimed to document and analyze the diversity, distribution, and sustainability of the traditional medicinal plants used by the Gurung people of the Sikles region in western Nepal. Ethnobotanical data were collected through focus group discussions and individual interviews, and analyzed using descriptive and inferential statistics. Prior informed consent was obtained before each interview. Quantitative ethnobotanical indices such as informant consensus factor, relative frequency of citation, and use values were also calculated. A possible association among these indices was tested using correlation analysis. A total of 115 wild medicinal plant species belonging to 106 genera and 71 families were documented. Asteraceae and Rosaceae were the dominant families whereas herbs were the most dominant life form. Roots were the most used plant part, paste was the most common method of preparation, and most of the medical formulations were taken orally. The highest number of medicinal plants were used to treat stomach disorders. The average informant consensus value of 0.79 indicates a high consensus among respondents in selecting medicinal plants. *Lindera neesiana*, *Neopicrorhiza scrophulariiflora*, *Paris polyphylla*, and *Bergenia ciliata* were found to be high-ranking medicinal plants based on the relative frequency of citation and use value. The genders did not affect medicinal plants’ knowledge but age had a significant correlation. Most of the informants agreed that medicinal plants are under pressure due to overharvesting and a lack of proper forest management practices. The number of medicinal plants reported from the study area indicates that the Gurung people possess rich traditional knowledge, and the vegetation of the Sikles region constitutes rich diversity of medicinal plants.

## Introduction

The cultural practices determine how plants are used (Teixidor-Toneu et al., 2018). Medicinal plants are one of the most diverse groups of plants used traditionally in different cultures worldwide (Uprety et al., 2010; Uprety et al., 2012; Abera, 2014; Aziz et al., 2018; da Costa Ferreira et al., 2021; Lin et al., 2021; Kunwar et al., 2022; Turpin et al., 2022). The interest in the traditional use of medicinal plants has shifted from the treatment and management of various diseases and ailments (Sheng-Ji, 2001; Qureshi et al., 2016) to the discovery of modern medicine (Heinrich et al., 2009; Pan et al., 2013). The study of the local knowledge regarding use, collection, classification, conservation, and management of plants, that is the science of ethnobotany, is the interest of various academic disciplines around the world (Martin, 1995; Ballick and Cox 1996). Ethnobotanical research in many parts of the world has helped to understand the relationship between the plants and the indigenous communities (Abbas et al., 2016; Kassa et al., 2020; Hosseini et al., 2021; Kutal et al., 2021; Kunwar et al., 2022). Such studies are primarily focused on enlightening significant indigenous plant species (Cox, 2000) and at the same time play a significant role in the protection of traditional knowledge and conservation of biodiversity (Turner et al., 2000; Leonti, 2011; Jessen et al., 2022).

Generally, ethnobotanical studies are conducted with the indigenous peoples and local communities where traditional knowledge is mostly left undocumented (Kunwar and Bussmann, 2008; Adhikari et al., 2019). However, such knowledge is decreasing rapidly around the globe mainly due to globalization, modernization, and market integration (McDade et al., 2007) resulting in an asymmetrical pattern of knowledge loss (Díaz et al., 2006; Aswani et al., 2018). In the context of Nepal, this knowledge is usually held by the Vaidhyas (traditional healers) and elderly people and transmitted to the next-generation *via* verbal communication as a major route (Kunwar et al., 2010; Uprety et al., 2016; Joshi et al., 2020). There is a risk of knowledge loss due to the progression of the modern healthcare system, rapid urbanization, and loss of biodiversity. Therefore, proper documentation and preservation of traditional knowledge of different ethnic groups is of urgent need and have to be given a high priority in the place where biodiversity loss is in alarming rate. Moreever, the role of traditional knowledge, indigenous communities, and ethnobotanists has to be recognized on an urgent basis in realizing the Sustainable Development Goals (Pei et al., 2020; Kumar et al., 2021). Traditional knowledge must be utilized to alleviate poverty, end hunger, provide better healthcare facilities, understand climate change, conserve biodiversity, and solve biodiversity related issues (Peters et al., 2012; Kumar et al., 2021).

Several socio-economic factors such as settlement in a particular area, population, gender, age, ethnicity, economy, and occupation have shown a greater effect on knowledge of plant uses (Reyes-García et al., 2006; Voeks, 2007; Kutal et al., 2021). Nepal is rich in biological diversity because of diverse topography and climatic variations harboring more than 5,500 species of flowering plants (Shrestha et al., 2022). Similarly, because of socio-cultural diversity, there is variation in the use of plants in the country (Manandhar, 2002; Uprety et al., 2022). Furthermore, mountain regions are mostly recognized as hotspots of ethnopharmacologically important species (Wambulwa et al., 2021). Local species diversity and the historic isolation of mountain settlements lead to unique ethnopharmacological knowledge supporting the healthcare of local communities (Petelka et al., 2020; Mehrnia et al., 2021).

Several ethnobotanical studies have been conducted in Nepal focusing either on a particular geographical area/district (Shrestha and Dhillion, 2003; Bhattarai et al., 2006; Kunwar et al., 2006, 2018; Rokaya et al., 2010; Malla et al., 2015; Shrestha et al., 2016; Joshi et al., 2020) or ethnic group (Uprety et al., 2010; Rijal, 2011; Luitel et al., 2014; Ambu et al., 2020; Joshi et al., 2020). Most of these studies focused on the documentation of medicinal plants, their use patterns, and methods, while some other studies also focused on evaluating the bio-efficacy of medicinal plants based on known phytochemical and pharmacological properties (Kunwar et al., 2009, 2010; Uprety et al., 2010). Recently, ethnobotanical studies in Nepal have shifted to incorporate conservation, management, and cultural significance (Kunwar et al., 2018; Rana et al., 2020). However, the sustainability aspects in terms of knowledge transfer and resource conservation are still little understood. In the context of the Gurung community, Coburn (Coburn, 1984) first studied the native medicinal plants of the western Gurungs. Other studies includes Gorkha (Manandhar, 1990; Shah et al., 2020) and Kaski (Kumar Rana et al., 2015; Gurung and Rajbhandary, 2017).

Traditional knowledge about the use of medicinal plants in the Gurung community plays an important role in local healthcare but has not been scientifically documented. In addition, traditional medicinal knowledge is threatened due to the lack of proper documentation and conservative inheritance patterns (Chen et al., 2016). Young people prefer to look for higher-income jobs in urban areas and are not interested in traditional medicinal knowledge (Kunwar et al., 2009; Arjona-García et al., 2021; Kutal et al., 2021). On the other hand, the status of the wild medicinal plant population is decreasing mostly because of overexploitation, habitat degradation, and invasive species (Chen et al., 2016; Kunwar et al., 2016; Howes et al., 2020). In this paper, we aimed at 1) documenting existing ethnobotanical knowledge on traditional medicinal plants in the Sikles region of western Nepal, 2) identifying important medicinal plant areas in the region, 3) finding local plant resources of particular interest for sustainable management, and 4) assessing the sustainability of traditional knowledge and wild medicinal plants.

## Materials and methods

### Study area

This study was carried out with the communities of Khilang, Parche, and Sikles villages of Madi Rural Municipality (Ward no. 1) in Gandaki Province of western Nepal. Sikles is the largest village, situated on the mountainside at an elevation of about 1,700 m. The Sikles area (combinedly called for all three villages) is a part of the Annapurna Conservation Area, which is the largest conservation area in Nepal. The area was selected because of its diverse culture and rich biodiversity (Khakurel et al., 2020, 2021). Secondly, comprehensive documentation of medicinal plants was lacking although previous studies covered a small part of the region (Kumar Rana et al., 2015; Gurung and Rajbhandary, 2017).

**FIGURE 1:**
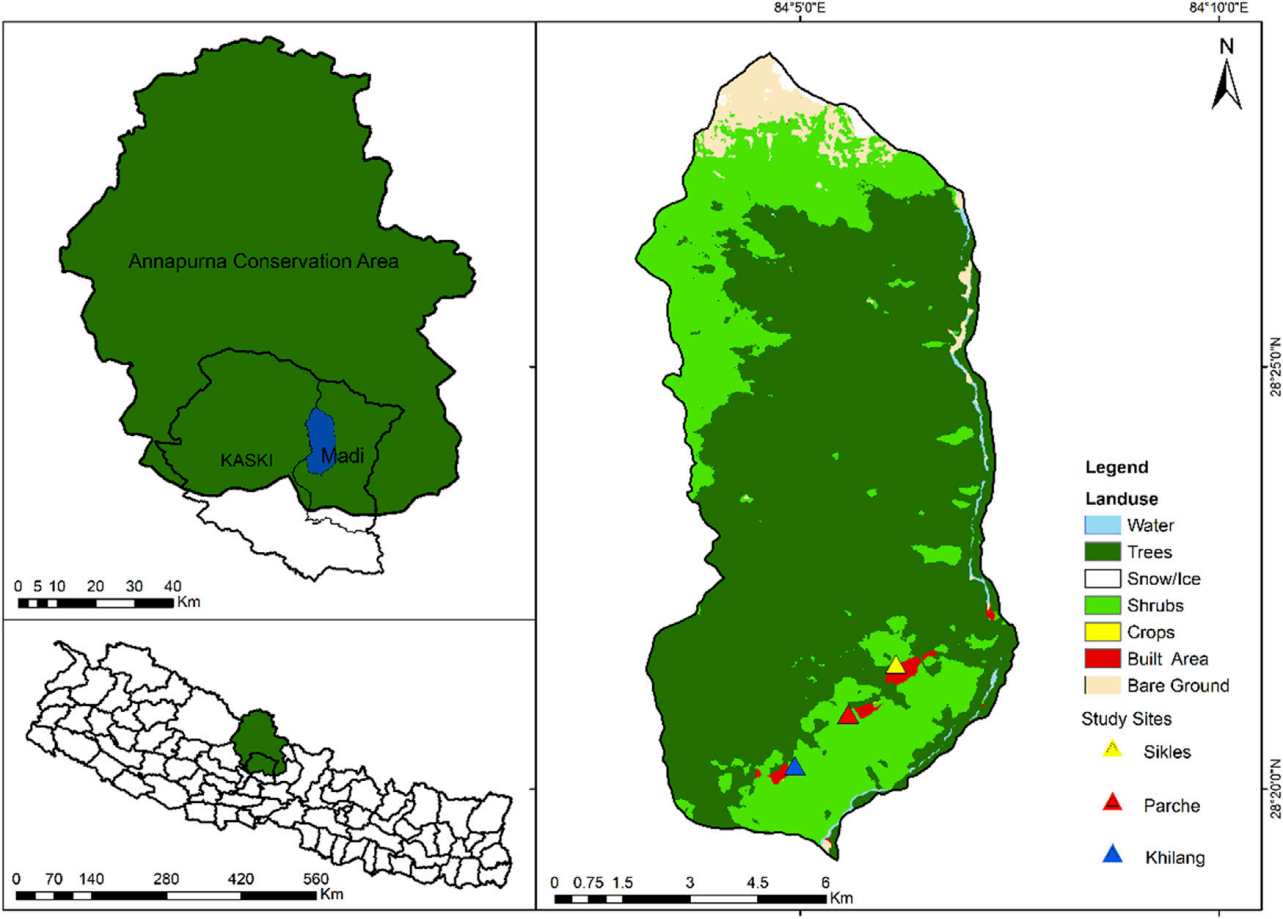
Map of the study area with land use pattern. The lower left is a map of Nepal with study district highlighted in green; the upper left is the map of Annapurna Conservation Area with Madi Rural Municipality in blue; and the map in right represents the exact location of the study area with land use pattern.

Geographically, Sikles lies between the upper subtropical to the lower alpine regions but mostly covers a temperate region. Altitudinal and climatic variations create high diversity of plants in the Sikles area which consist of upper subtropical to lower alpine vegetation (Figure 1). *Alnus* forests, mixed forests, broad-leaved forests, bushes, and high-altitude grasslands are available in the region. This area is characterized by typical assembles of both western and eastern Himalayan floral elements (Khakurel et al., 2020).

A total population of all three villages is about 2,418, among which Gurungs account for 70% (CBS, 2011). The Gurungs are one of the major ethnic groups of Nepal traditionally lived in mid-to high-hills possessing many generations of experience with the local vegetation. This community has a unique adaptation to different environmental conditions, as revealed by their culture and livelihoods (Messerschmidt, 1976). Agriculture is the main livelihood strategy adopted in the area. In addition, nature-based tourism has contributed largely in local livelihood.

### Methods

#### Prior informant consent

The research objectives were discussed and permissions were obtained from the Department of National Parks and Wildlife Conservation, Annapurna Conservation Area Project, and the local government authority. Prior oral informed consent for recording and disseminating the traditional knowledge was obtained from the participants of the study. The communities were further ensured that the access and benefit sharing (ABS) process and laws would apply in the case of further research and development as the study area was one of the pilot sites for the ABS project of the Government of Nepal and IUCN (Uprety et al., 2020).

#### Sampling design and informant selection

An ethnobotanical reconnaissance visit was first done in January 2018 and data collection was conducted in April and August 2018. Village heads, traditional healers, medicinal plant collectors, herders, and housewives were invited to participate in the Focus Group Discussions (FGDs). Participants were randomly selected, irrespective of age, occupation, sex, religion, and education level for interviews (Martin, 1995; Cunningham, 2001; Title et al., 2014).

#### Focus group discussions

A total of five FGDs were conducted one each in Khilang and Parche, and three in Sikles. About 7–10 participants representing traditional healers, medicinal plant collectors, housewives, elderly people, and other local people from different gender and age groups participated in each group discussion. The participants were selected based on their rich indigenous knowledge and long-term experience on the utilization of plants as well as their living period in the study area as recommended by the local administration and staff of the Annapurna Conservation Area Project (ACAP) office. Ethnomedicinal information such as uses, mode of use, parts used, and local name(s) were documented during the FGDs. Moreover, the perceived status of the species and the areas rich with medicinal plants were also documented during the FGDs and this information was triangulated in a participatory resource mapping exercise followed by field observation and transect walk with the selected participants. The checklist of the useful plants was prepared during discussions. Finally, ten key informants were selected from the FGDs for detailed information about the use of medicinal plants and four participants were selected for field observation and transect walk. Also, a small group discussion (*n* = 14) with adolescence (12–18 years) was conducted to find knowledge about medicinal plants among themselves.

#### Key informant interviews

A total of 70 informants (40 males and 30 females) including 10 key informants (6 males and 4 females) between the age of 20 and 72 were interviewed for detailed information about the traditional use of medicinal plants. For collection and recording of indigenous knowledge held by certain social groups, the choice of key informant is crucial (Scherrer et al., 2005). Accordingly, at least two key informants were selected from each site using a purposive sampling technique ensuring that the knowledgeable persons are included in the pool. The key informants include a head of the mother group, two housewives, a social worker, a person worked for ACAP, and five people practicing herbal medicines. The interviews with local people were done where they feel comfortable following semi-structured questionnaires. Since key informants had good knowledge on medicinal plants the detailed information about those plants, processing, cured disease, and parts used were gathered from them.

#### Transect walk

Transect walks were done with four people from 1800 m to 4,200 m in different seasons and areas to collect and identify the plants cited by the local people. The methods and the major areas for the collection of cited species were documented during these walks.

#### Plant specimen collection, identification, and nomenclature

Voucher specimens were collected during the transect walks. Field notes were taken to document relevant taxonomic characteristics for further taxonomic determination. The identification of plant species was done by consulting experts and standard literature (Polunin and Stainton, 1984; Stainton, 1987; Press et al., 2000). Comparison was also done with specimens deposited at the National Herbarium (KATH) and Tribhuvan University Central Herbarium (TUCH) to ensure taxonomic determination. The nomenclature follows The Plant List (http://www.theplantlist.org/). The voucher specimens were deposited at TUCH. Plant species were classified into native and exotic categories according with their biogeographical origin.

#### Data analysis

Both qualitative and quantitative methods were used to analyze data. Gender and age were used for the analysis of the sociocultural effect on ethnobotanical information. One-way ANOVA was performed between gender and number of medicinal plants reported to be used. Also, mean and standard deviation of number of plants reported by both genders were calculated. The regression analysis was done on participant’s age and number of medicinal plants reported by them. Analysis was carried out using SPSS version 23.00. The quantitative ethnobotanical tools such as Informant Consensus Factor (ICF or Fic), Use Values (UV) and Relative Frequency of Citation (RFC) were also used for data analysis. A possible association among these indices was tested using correlation analysis.

##### Informant consensus factor

Informant Consensus Factor (Fic) was used to identify the potentially effective medicinal plants (Trotter and Logan, 1986). This method identifies groups of plants requiring more in depth study (Heinrich et al., 1998; Andrea-Cetto and Heinrich, 2011). Fic gives agreement of local people on the use of plant for particular ailments categories. The Fic values range from 0 to 1 and a high value of Fic (close to 1) is obtained only when one or a few plant species are reported to be used by high proportion of informants to treat a particular ailment.

The Fic was calculated following Trotter and Logan (1986), Heinrich et al. (1998) as:

$$Fic=\frac{Nur-Nt}{Nur-1}$$

Where, ‘Nur’ is the number of use reports in each ailment category and ‘Nt’ is the total number of taxa used in each ailment category by all the informants.

##### Use value

Use value (UV) was calculated in order to find out the relative importance of medicinal plant species following Phillips and Gentry (1993):

$$UV=\frac{Uc}{N}$$

Where, ‘Uc’ is the number of use reports cited by each informant for a given plant species and ‘N' is total number of informants interviewed for the given plant species.

##### Relative frequency of citation

Relative frequency of citation (RFC) shows how frequently a particular plant species is used at the local level. High RFC is obtained when many informants cite the given species and low value is obtained when a few informants cite the given species.

RFC was calculated following Tardío and Pardo-De-Santayana (2008) as:

$$RFC=\frac{U}{N}$$

Where, ‘U' is number of informants who mentioned the use of given plant species and ‘N' is the total number of informants interviewed.

## Results and discussion

### Demography and knowledge distribution among respondents

Average age of the informants was 47 with 72 maximum and 21 minimum. The average years of experience of using one or more medicinal plants among informants was 14 while 45 was maximum. Since subset of socioeconomic factors such as gender, age, length of residence in the particular area, and occupation play crucial role in the knowledge acquisition about medicinal plants (Voeks, 2007; Kutal et al., 2021), it is important to understand these factor while collecting information.

We used gender as a first classification criterion to understand whether the knowledge regarding medicinal plants varies among them and how this variable influences the structure of local medical systems. Here, the one-way ANOVA demonstrated that the number of plants listed by women and men informants was not significantly different (*p* = 0.1023) although, on average, more medicinal plants were reported by male informants (12.325 ± 3.725) than female informants (10.923 ± 2.425) (Mean ± S.D.). Similar results were obtained by Joshi et al. (2020) in Makawanpur (Nepal) and Bibi et al. (2022) in Himalayan region of Pakistan, where gender had no influence on medicinal plants knowledge. Torres Avilez et al. (2016) in their meta-analysis revealed no significant difference between the genders although there was significant difference in country and continent levels. On the other hand, women stands more with method of preparation and mode of administration of different medicinal plants. This dissimilarity between genders may be due to the fact that men generally manage the fieldwork and earning and travel high altitude for medicinal plant collection while women manage mostly the indoor activities and domestic life, which are highly associated with herbal preparations and uses.

**FIGURE 2:**
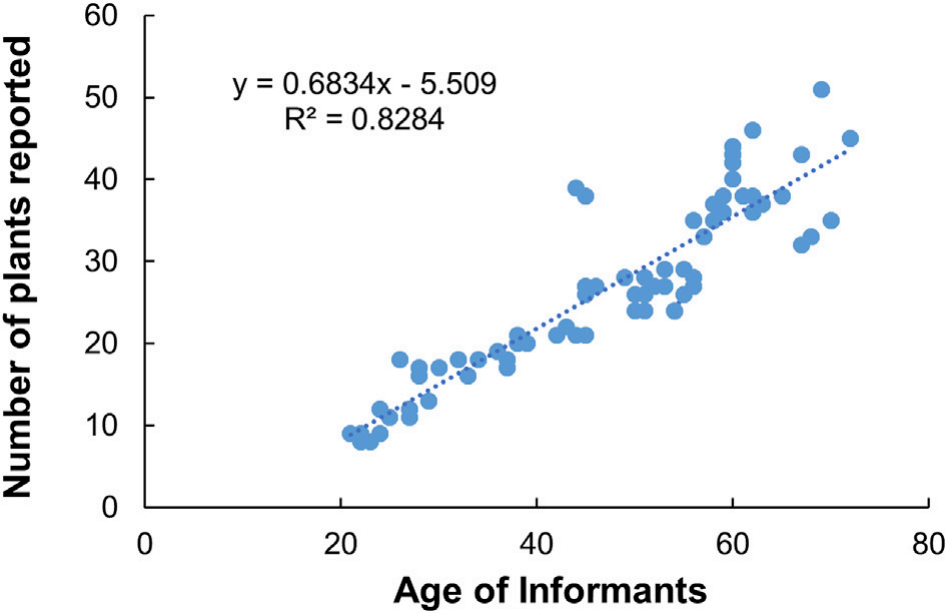
Relationship between participants’ knowledge and age.

Age was used as second classification criterion to find out knowledge distribution among informants. Our result shows that the knowledge of the participants and age had a positive significant correlation (*R* ^2^ = 0.82, *p* < 0.05) (Figure 2). Thus, elder people tended to know more about medicinal plants than their younger counterparts. Particularly, age between 45 and 65 years cited higher number of medicinal plants and their uses comparing to other age groups.

Similar pattern of knowledge distribution is reported from Nepal (Rijal, 2008; Luitel et al., 2014), Mexico (Benz et al., 2000), Bolivia (Reyes-García et al., 2006), Ethiopia (Yineger et al., 2008), and Pakistan (Amjad et al., 2020). Reasons for greater knowledge with increasing age could be numerous and have to be interpreted with caution (Al-Fatimi, 2019; Weckmüller et al., 2019). It is logical to mention that with progressive age, people have more time to accumulate knowledge (Voeks and Leony, 2004). Discussion session with young people (*n* = 14 and average age 16 years) showed that they could name only a few medicinal plants. Other information regarding use, collection sites, and preparation methods was restricted to a very few participants. This demonstrates the gap in knowledge transmission, a common threat to traditional knowledge observed elsewhere (Geissler et al., 2002; Bruschi et al., 2019; Sousa et al., 2022).

### Diversity of medicinal plants

A total of 115 wild medicinal plant species belonging to 106 genera and 71 families were reported in this study. Among them nine species were pteridophytes, two species were gymnosperms and remaining 104 species were angiosperms (Supplementary Table S1). The present report of 115 medicinal plant species is about 38% of the total plant species reported from the study area (Khakurel et al., 2020). Uprety et al. (2011) reported only 24% of total flora used as medicinal in Tanahun district. This indicates that the area is an excellent reservoir of plant species of medicinal value. According to Giday and Teklehaymanot. (2013) high diversity of medicinal plants is attributed to good vegetation cover which in turn implies their significant role in plant-based traditional medicine in meeting basic primary healthcare needs. Comparing to the previous study in the same area conducted by Kumar Rana et al. (2015) who reported 42 species and Gurung and Rajbhandary (2017) who reported 66 species, our study reported relatively high number of medicinal plant species with some new reports. Both of the previous studies covered only a small part of the study area but this study covered larger area, included more participants and also covered different seasons. Regarding the origin of plants most of the species were naturalized species with few exotic species as *Ageratum conyzoides* L. [Asteraceae], *Cynoglossum zeylanicum* (Vahl) Thub. ex Lehm. [Boraginaceae] *etc.*

### Life forms

Of the total species reported, 65 (58%) were herbs followed by 24 (21%) trees, 15 (13%) shrubs, and 10 (8%) climbers. This distribution pattern in different life forms is similar to other studies from Nepal (Shrestha and Dhillion, 2003; Kunwar et al., 2006; Uprety et al., 2010). The dominance of herbs could be explained by the fact that the study sites are located in higher elevations ranges thus diversity of herbs or shrubs is higher than that of trees. Use of shrub is, however, less common than use of trees for medicinal purposes (Rokaya et al., 2010). In fact, the study area harbors highest number of herbs (Khakurel et al., 2020). Asteraceae and Rosaceae were the dominant families with six species each followed by Gentinaceae and Ranunculaceae with four species each. Similarly, Berberidaceae, Asparagaceae, Polygonaceae, Lamiaceae, Solanaceae, Apiaceae, and Orchidaceae had three species each. Similar accounts of over utilization of these families are common in different parts of Nepal (Kunwar and Bussmann, 2008; Rokaya et al., 2010; Ambu et al., 2020; Kutal et al., 2021; Kunwar et al., 2022).

### Parts used

**FIGURE 3:**
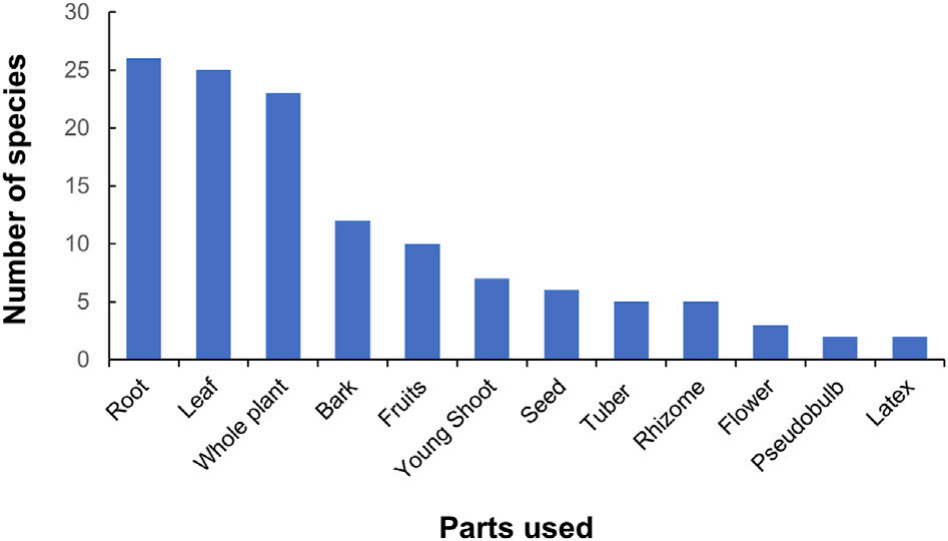
Plant parts used for medicinal use.

Altogether 13 parts (including latex) of the plants were used to treat different diseases. In case of some species multiple parts were used (13 species). Roots (22.80%) were the most used plant parts followed by leaves (21.93%), whole plant (20.18%), and bark (10.53%) (Figure 3). The whole plant was also used, mostly in the case of herbs. Generally, underground parts (roots/rhizomes/bulbs/tubers) were most frequently used. These results show consistency with the other ethnobotanical studies in the Himalayan regions of Nepal (Rokaya et al., 2010; Uprety et al., 2010). Underground parts of plants are the most preferred parts for medicinal purposes possibly because they contain higher amount of bioactive compounds than other parts (Srithi et al., 2009). Excessive use of reproductive parts and roots for the medicine has a negative effect to the population of plants resulting in the declining plant population (Giday and Teklehaymanot, 2013).

### Method of preparation and administration

According to the respondents, there are certain medicinal plant species that should be collected on specific time or day for their high medicinal efficacy. For instance, *Dactylorhiza hatagirea* (D. Don) Soó [Orchidaceae], *Aconitum gammiei* Stapf [Ranunculaceae] and *Hymenidium benthamii* (Wall. ex DC.) M.G. Pimenov & E.V. Kljuykov [Apiaceae] were collected only on Tuesday and respondents believe that the tuber collected from these species on Tuesday had effective medicinal property.

**FIGURE 4:**
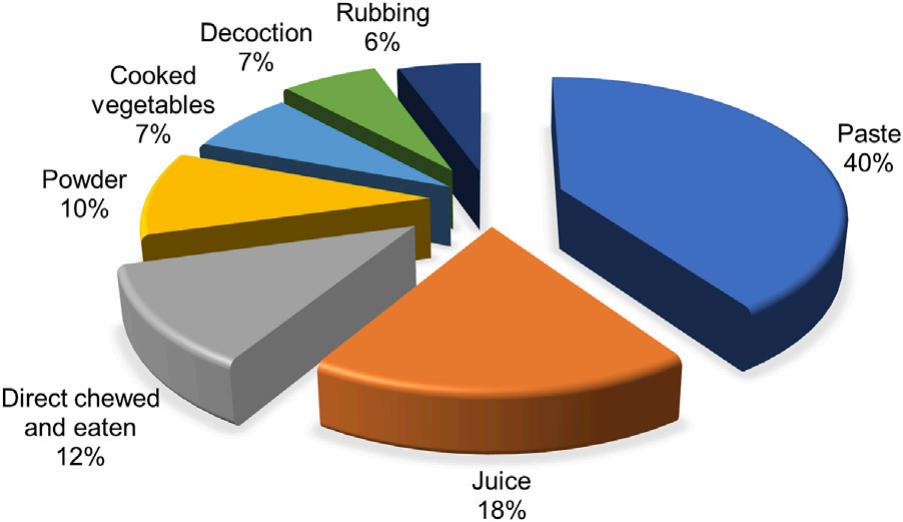
Mode of administration of medicinal plants.

The methods of preparation and administration of medicinal plants were diverse. Some formulations were prepared using single species (92 species) while others were prepared using multiple species (22 species). For example, the paste prepared from the stem of *Rheum australe* D. Don [Polygonaceae], *Dendrobium amoenum* Wall.ex Lindl. [Orchidaceae], and *Prunus cerasoides* D. Don [Rosaceae] were used in bone fracture. Rhizome of *Acorus calamus* L. [Acoraceae] was chewed to get relief from cough and throat pain. A cooked vegetable prepared from young shoot of *Phytolacca acinosa* Roxb. [Phytolaccaceae] was used to cure stomachache and diarrhea. Paste (40%) was the most common preparation followed by juice (18.23%), powder (10%), and decoction (7%) (Figure 4). Some 12% species were also chewed or eaten directly. Paste, powder and juice are the popular methods of preparation of medicinal plants in other parts of Nepal (Rokaya et al., 2010; Uprety et al., 2010).

The most common mode of administration of medicine is oral (65%, *n* = 75), followed by external application (32%, *n* = 31), and inhalation (4%, *n* = 4) as also reported by (Bhattarai et al., 2010; Luitel et al., 2014).

### Ailments treated

Medicinal plants were used to treat 23 different ailments. Highest number of species (22) were prescribed in stomach problems (abdominal pain, indigestion, gastric, constipation, intestinal problems) followed by 18 in fever, 16 in skin problems (mumps, scabies, and small wounds), 13 each in cut and wounds, burns, and diarrhea and dysentery (Table 1). Regarding reliability of the use reports, the use of the plants obtained in our studies compared with the ethnobotanical publications of Nepal shows a good accordance (Coburn, 1984; Lama et al., 2001; Manandhar, 2002; Shrestha and Dhillion, 2003; Baral and Kurmi, 2006; Bhattarai et al., 2006; Kunwar et al., 2006; Kunwar and Bussmann, 2008; Rokaya et al., 2010; Uprety et al., 2010; Malla et al., 2015; Gurung and Rajbhandary, 2017; Adhikari et al., 2019). This similarity is of great significance because identical plant use by different people from different areas may also be a reliable indication of curative properties (Uprety et al., 2010). For example, different previous studies shows that *D*. *hatagirea* is used for stomach problems which was consistent with the current study. Similarly, *Swertia chirayita* (Roxb.) H. Karst. [Gentianaceae] previously reported to cure fever, headaches and stomach problems which is also good accordance with the current results.

**TABLE 1:** Informant consensus factor and different ailments category.

Disease/ailments	Number of taxa (Nt)	Number of use reports (Nur)	Informant consensus factor (Fic)	Name of species^a^
Stomach problems (Abdominal pain, Indigestion, gastric, constipation, intestinal problems)	22	77	0.72	*Tectaria coadunata, Chenopodium album, Artemisia indica, Begonia picta, Cannabis sativa, Solena amplexicaulis, Elaeagnus parvifolia, Rhododendron arboreum, Swertia chirayita, Lindera neesiana, Fritillaria cirrhosa, Paris polyphylla, Pedicularis siphonantha, Neopicrorhiza scrophulariiflora, Aconitum gammiei, Delphinium vestitum, Argentina lineata, Fragaria nubicola, Rubus ellipticus, Zanthoxylum armatum, Girardinia diversifolia, Dactylorhiza hatagirea*
Fever	18	76	0.77	*Alsophila spinulosa*, *Nephrolepis cordifolia, Aleuritopteris rufa, Achyranthes aspera, Cirsium verutum, Berberis concinna, Cuscuta reflexa, Solena amplexicaulis, Dioscorea deltoidea, Swertia angustifolia, Swertia chirayita, Swertia paniculata, Colebrookea oppositifolia, Cinnamomum tamala, Neopicrorhiza scrophulariiflora, Aconitum gammiei, Thalictrum foliolosum, Urtica dioica*
Skin problems (*Goda futeko,* mumps, scabies, small wounds)	16	72	0.79	*Lygodium japonicum*, *Selaginella pennata*, *Taxus wallichiana, Artemisia indica, Lyonia ovalifolia, Juglans regia, Lycopodium clavatum*, *Leucosceptrum canum, Reinwardtia indica, Paris polyphylla, Coelogyne cristata, Rumex nepalensis, Maesa chisia, Pyrularia edulis, Rubia manjith, Smilax aspera, Viola pilosa*
Cut and Wounds, Burns	13	58	0.79	*Equisetum arvense, Aleuritopteris albomarginata, Ageratina adenophora, Ageratum conyzoides, Taraxacum officinale, Cyanoglossum zeylanicum, Dipsacus inermis, Bombax ceiba, Ficus auriculata, Fragaria nubicola, Galium elegans, Houttuynia cordata, Solanum nigrum*
Diarrhea and dysentery	13	73	0.83	*Aleuritopteris rufa, Berberis napaulensis, Rhododendron arboreum, Erythrina arborescens, Morella esculenta, Phytolacca acinosa, Plantago major, Rheum australe, Thalictrum foliolosum, Pyracantha crenulata, Astilbe rivularis, Bergenia ciliata, Brucea javanica*
Cough and Cold (Sore throat, Sinusitis)	12	58	0.82	*Juniperus squamata, Acorus calamus*, *Saurauia napaulensis*, *Centella asiatica*, *Hymenidium benthamii*, *Drymaria cordata*, *Rhododendron arboreum*, *Swertia paniculata*, *Cinnamomum tamala*, *Fritillaria cirrhosa*, *Piper mullesua*, *Fragaria nubicola*
Sprain (Joint pain, Body pain, Muscle pain, gout)	11	35	0.71	*Choerospondias axillaris, Ligusticopsis wallichiana, Ligusticopsis wallichiana, Agave americana, Anaphalis contorta, Euphorbia royleana, Quercus lanata, Phyllanthus parvifolius, Rheum australe, Prinsepia utilis, Urtica dioica*
Fracture (Bone problems, external and internal injury)	8	27	0.73	*Choerospondias axillaris, Elaeagnus parvifolia, Dendrobium amoenum, Rheum acuminatum, Rheum australe, Prunus cerasoides, Schima wallichii, Curcuma angustifolia*
Tonic (give strength to body)	4	13	0.75	*Alsophila spinulosa*, *Allium wallichii*, *Asparagus racemosus*, *Polygonatum cirrhifolium*
Jaundice (Including Typhoid)	4	16	0.80	*Rubus ellipticus*, *Achyranthes aspera*, *Berberis aristata*, *Cuscuta reflexa*
Headache	4	17	0.81	*Centella asiatica, Drymaria cordata, Rheum australe, Dactylorhiza hatagirea*
Urinary problems	3	6	0.60	*Berberis napaulensis*, *Plantago major*, *Cynodon dactylon*
Eye Problem	3	7	0.67	*Maharanga emodi*, *Oxalis corniculata, Tetrastigma serrulatum*
Respiratory Problems	3	5	0.50	*Lobelia pyramidalis*, *Morella esculenta*, *Piper mullesua*
Menstrual Problems	2	4	0.67	*Euphorbia royleana*, *Nardostachys grandiflora*
Vomiting	2	4	0.67	*Trichosanthes tricuspidata*, *Lindera neesiana*
Toothache	2	6	0.80	*Argentina lineata*, *Solanum aculeatissimum*
Post-natal Recovery	2	5	0.75	*Astilbe rivularis*, *Bergenia ciliata*
Lactation (Increase milk in post-natal mother)	1	3	1.00	*Asparagus racemosus*
Worm	1	5	1.00	*Maharanga emodi*
Repellent	1	4	1.00	*Elsholtzia blanda*
Dandruff	1	8	1.00	*Rumex nepalensis*
Ear problems	1	4	1.00	*Nicotiana tabacum*

^a^ Complete author names of each plant species and their respective family were annotated in Supplementary Table S1.

Empirical observation on the use of medicinal plants by the *Gurung* people on the Kaski district needed to be substantiated with phytochemical and pharmacological studies in order to corroborate their bio-efficacy. Comparisons of local uses and phytochemical/pharmacological properties for most of the medicinal plants showed that traditional use was coherent with known pharmacological and phytochemical properties in most of the cases. For example, *Artemisia indica* Willd. [Asteraceae] is reported to use for skin problems form our study, phytochemical investigation shows that it has antimicrobial properties (Rashid et al., 2013). Similarly, *Paris polyphylla* Sm. [Melanthiaceae] is used for the stomach problems. Phytochemical investigations shows that its methanolic extract is gastro-protective as well as anthelmintic properties (Matsuda et al., 2003). In addition, *Bergenia ciliata* (Haw.) Sternb. [Saxifragaceae] has multiple used mainly in gastrointestinal problems and skeletal problems, pharmacological investigations revealed that it contains antioxidant and anti-inflammatory properties (Ahmad et al., 2018; Zafar et al., 2019). The major aim of the ethnobotanical field studies is to provide main plants of the certain region to perform further phytochemical and pharmacological studies (Andrade-Cetto, 2009). Our study also provide numerous plant species which shows important by quantitative data promising for further phytochemical investigations. Species with high use value but less pharmacologically investigated need to focus for further study. Species such as *Pedicularis siphonantha* D. Don [Orobanchaceae]*, Polygonatum cirrhifolium* (Wall.) Royle [Asparagaceae], *Swertia paniculata* Wall. [Gentianaceae], and *Tectaria coadunata* (J. Sm.) C. Chr. [Tectariaceae] needs further attention.

By comparing the recorded medicinal plants from this study with the other research and database of Nepal, we found that two species namely *Berberis concinna* Hook. fil. [Berberidaceae] (used for fever), *Hymenidium benthamii* (Wall. ex DC.) M.G. Pimenov & E.v. Kljuykov [Apiaceae] (for cough and cold) have not been reported previously as medicinal plants. Detail phytochemical investigations seems promising for these species.

### Distribution of medicinal plants

**FIGURE 5:**
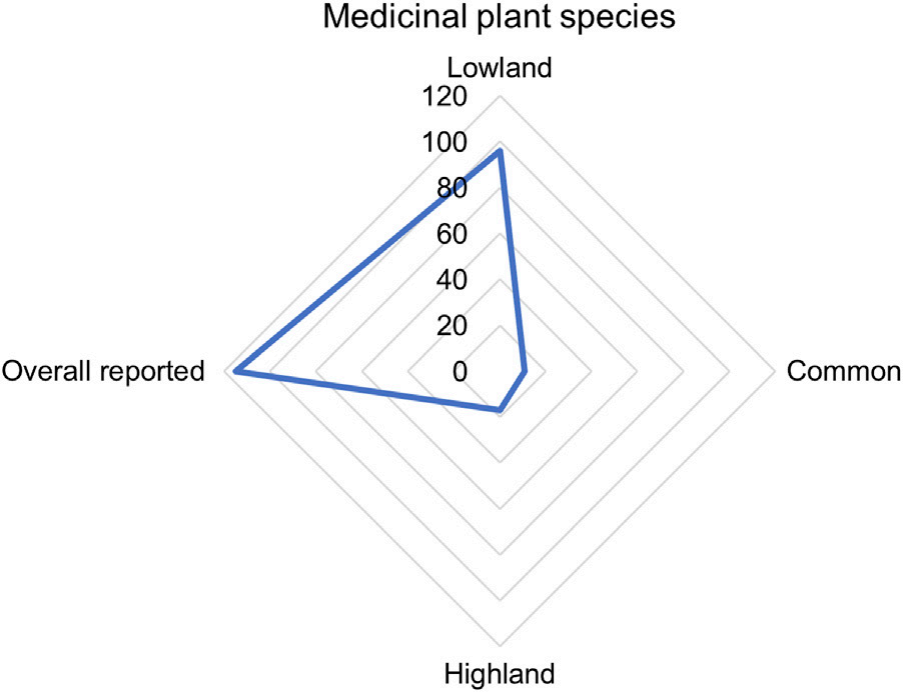
Distribution of medicinal plant species in different areas (Sum exceed 115 because some species can be collected from more than one habitat).

Forests, fallow lands, and grasslands were predominant collection sites for the medicinal plants. Of the total species, 89 species were reported to be collected from forests followed by 21 species from grasslands, 10 species from fallow land, 4 species from roadside, and 3 species from riverside. Some specific plants are habitat specific such as *B*. *ciliata* (H is found in rocky areas in lowland while *Neopicrorhiza scrophulariiflora* (Pennell) D.Y. Hong [Plantaginaceae] is found in the rocky slopes in high altitude. The habitat of the medicinal plants can be divided into the two parts in terms of collection time and altitude - lowlands (between 1800 m and 3,000 m; near and around villages, collection time within a day, collection areas easily accessible) and highlands (above 3,000 m; far from villages, collection takes more than a day, collection areas remote and hard to access). Out of the reported species, 96 species are found in the lowlands and 17 species were found in the highlands with 10 species common in lowlands and highlands (Figure 5). Generally, collection of highland medicinal plants is difficult and hence storage of such species for further use is common practice in the study area.

Collection of most of the medicinal plants from wild habitats is a common practice in Nepal (Kunwar et al., 2006; Rokaya et al., 2010; Luitel et al., 2014; Shrestha et al., 2016; Uprety et al., 2016) and elsewhere (Pieroni, 2000; Abbas et al., 2016; Singh et al., 2017; Kassa et al., 2020). Many medicinally important plant species were reported to be found in lowlands near villages as these are mid-altitudinal ranges (1,700–2,200 m). This is in line with Kunwar and Bussmann (2008) who reported an increase of medicinal plant species with increasing altitude up to about 2000 m. The medicinal plant diversity corresponds with total richness of plant diversity in Nepal (Bhattarai and Vetaas, 2003), however, the high value species were reported from the highland areas.

#### Informant consensus factor and use values

We obtained Fic values from 0.5 to 1.00 with average of 0.79 indicating thast most of the informants agreed in the use of specific medicinal plants to treat a particular ailment. Use of plants to treat ear problems, dandruffs, intestinal worms, and to increase lactation had highest Fic values followed by diarrhea and dysentery and cough and cold. A high value reflects high dependence of local inhabitants on local medicinal plants and low values indicate less consistency of informant’s knowledge (Heinrich et al., 1998). Fic values are generally influenced by the number of informants and are more significant when calculated for uses cited by many informants (Heinrich et al., 2009; Andrea-Cetto and Heinrich, 2011). In general, Fic values were high for most of the ailments in our study revealing that most of the informants tend to agree on the use of particular plant species. High Fic can help in identifying potentially effective medicinal plants (Teklehaymanot and Giday, 2007; Heinrich et al., 2018). We observed that the highest agreement level was recorded for diseases reported as the most widespread in local communities of different parts of Nepal (Rokaya et al., 2010; Uprety et al., 2010; Adhikari et al., 2019).

The highest value of RFC ranked the *S*. *chirayita* (0.84) first, followed by *N*. scrophulariiflora (0.80) and *P*. *polyphylla* (0.77) (Supplementary Table S1). The species with high RFC value were abundant in the area therefore most of the local people were familiar with them. Likewise, the plants with special properties to cure particular disease were well known among the local culture; therefore, their precise properties to treat particular disease have got famous and deep rooted. The plant species with high RFC values would be interesting for further research. Studies suggest that higher RFC values indicate the retention and smooth transmission of traditional knowledge among local people (Tounekti et al., 2019).

Use value ranged between 3.07 and 0.60 in the present study (Supplementary Table S1). The plant species with high use values were *P*. *polyphylla* (3.70) followed by *L*. *neesiana* ((3.05), *R*. *australe* (2.84), *B*. *ciliata* (2.54), *Astilbe rivularis* Buch.-Ham. ex D. Don [Saxifragaceae] (2.22), *A*. *calamus* (2.21), *N*. *scrophulariiflora* (2.18) and *D*. *hatagirea* (2.13). The ethnobotanical research assumes that the more often a plant is reported to be useful the more often it is going to be used (Bekalo et al., 2009). UV is directly related with use reports. Plant species with more use reports have high use value and less use have less use value (Zenderland et al., 2019). Most of the plant species in the present study with high use values have multiple uses. For example, *P*. *polyphylla* was used in cut, wounds, burns and stomach pain. Similarly, *L*. *neesiana* and *N*. *scrophulariiflora* were extensively used in stomach problems and fever. Species with high RFC and UV show high healing potential for particular disease (Cordero et al., 2022). Species with high RFC and UV were often overharvested by inhabitants, so they should be prioritized for conservation and sustainable use; otherwise, they will be extinct from the area in the near future (Chen et al., 2016; Amjad et al., 2020). Although plants with high RFC or UV are the most preferred species in study sites plants with low RFC or UV should not be neglected as failing to mention them to the future generation could increase the risk of gradual disappearance of the knowledge. Subjective intrepreation of the field collected ethnobotanical information along with careful quantitative analysis of data is crucial to identifying most important medicinal plant species (Andrea Cetto and Heinrich, 2011).

#### Relationship between relative use frequency and use value

**FIGURE 6:**
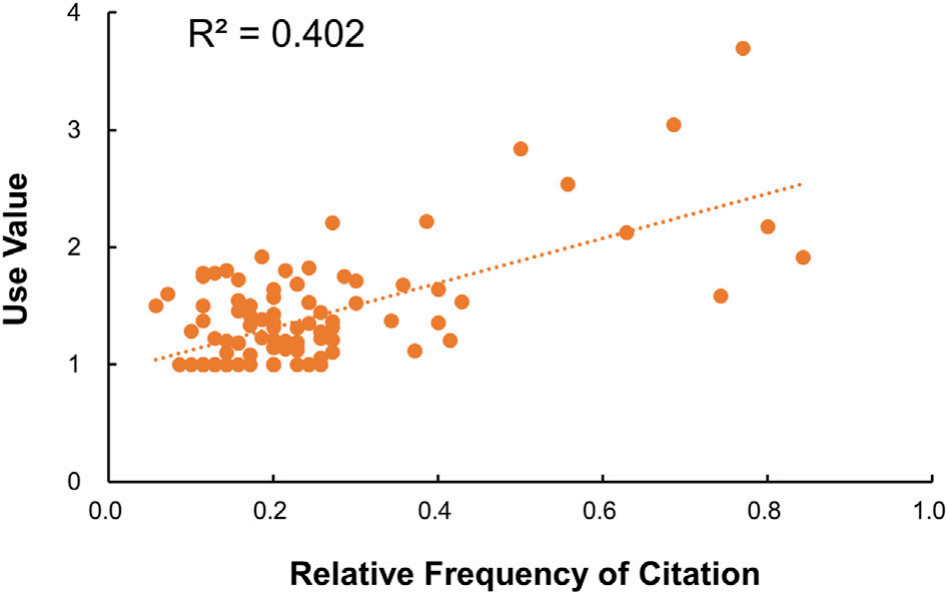
Correlation between RFCs and UVs.

The Pearson correlation coefficient between RFCs and UVs was 0.63 (*p*-value <0.05) indicating a high positive significant association between the local importance of each species and relative importance of the use of plants (Supplementary Tables S2, S3). It implies that more use of species by the informants tend to increase the number of usable medicinal plants. The fact that RFCs and UVs are strongly correlated means that their use pattern across species match (Bano et al., 2014). However, some species may have high RFC and UV while others have low values but have high importance in local level (Ahmad et al., 2017; Najem et al., 2019). The degree to which RFC and UV varies across species was measured numerically by R-squared which states that around 40% variation in RFC can be explained by that of UV. On the other hand, RFC does not indicate the diversity in plant utilization from the medicinal perspective and it can only be predicted by UV application (Bibi et al., 2022). Thus, the findings imply the empirical robustness among these two indices indicating that this work has significant contribution in the documentation of ethnobotanical information on medicinal plant uses. These findings are further supported by the scattered plot which reflects a strong positive relationship between RFCs and UVs (Figure 6).

### Knowledge about toxicity

Many plants used in traditional medicine or food have demonstrated some toxicity (Hammad et al., 2019). Nevertheless, plants used in traditional medicines have been considered safe as a result of the long history of use in the treatment of diseases based on knowledge accumulated over several centuries. In many cultural settings, toxic fatalities have been rare due to systematic selection of medicinal plants (Fennell et al., 2004). In our study also, local people had extensive knowledge about differentiating toxic and non-toxic medicinal plants on the basis of morphology or phenology of the species. For example, species from genus *Aconitum* and *Arisaema* have both medicinal and poisonous species which is distinguished by the local people. Generally, medicinal plants contain bioactive compounds which demonstrate both intra- and inter-species variation in type and content (Vaou et al., 2022).

Besides, studies documenting traditional knowledge about the use of medicinal plants rarely contain information on potential toxicity of the plants. This could be because such studies tend to focus more on the therapeutic property rather than on toxicological information (Botha and Penrith, 2008). This failure to document and contextualize potential toxicity of the plants in healing traditions and healing practitioners’ methods and approaches to treatment does not promote the safe use of medicinal plants outside the cultural boundaries (Rasool et al., 2022).

### Sustainability of medicinal plants

**FIGURE 7:**
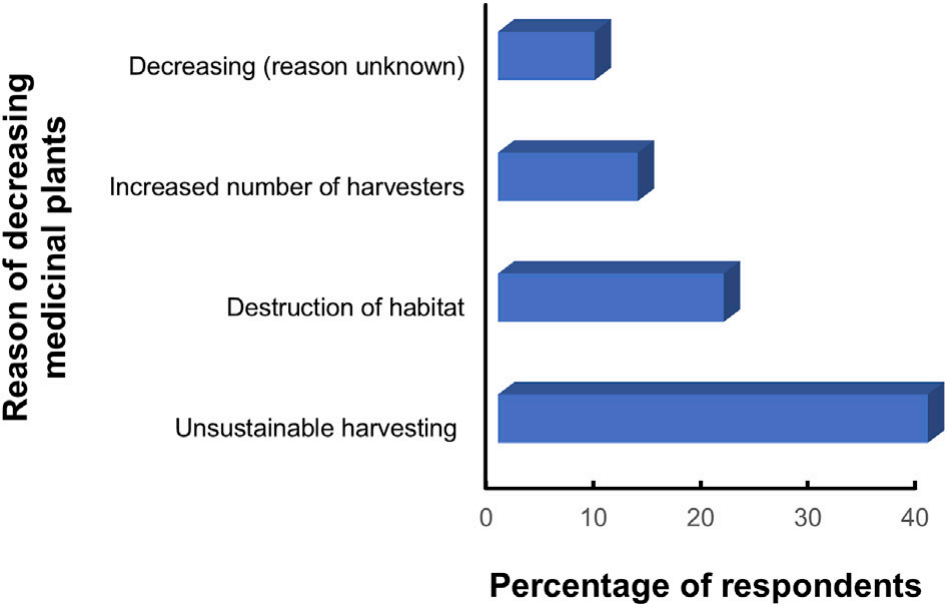
People perception on medicinal plant status.

More than 78% of the respondents mentioned that the population of the wild medicinal plants is declining while 15% were unaware and the rest mentioned that the status of medicinal plants is unchanged. Majority of the informants (40%) pointed out that the unsustainable harvesting including premature collection to meet trade demand is the major cause for decreasing population in the wild. Species with high traded values such as *A*. *gammiei*, *Asparagus racemosus* Willd. [Asparagaceae], *L*. *neesiana*, and *N*. *scrophulariiflora* and *D*. hatagirea were included in this category (Figure 7). Destruction of the habitat including deforestation and habitat fragmentation was second major factor responsible for decreasing the wild population of the medicinal plants. Other causes mentioned by the respondents were lack of proper forest management system and climate change. Overexploitation, increased harvesters, indiscriminate collection, uncontrolled deforestation, and habitat destruction are major threats for medicinal plants around the globe including in Nepal (Bhattarai et al., 2006; Kunwar and Bussmann, 2008; Abbas et al., 2016; Chen et al., 2016). However, it is important to note that not all medicinal plants are affected in the same way by harvesting pressures (van Andel and Havinga, 2008), some plants having multiple biological characteristics such as habitat specificity, distribution range, population size, species diversity, growth rate, and reproductive system play crucial role in the plants availability (Wagh and Jain, 2013; Chen et al., 2016). Root, leaves, and tubers play vital role in the life cycle of the plants, therefore, overharvesting of these parts should be minimized. There is a need for quantitative estimation of threatened species and such species require immediate action for conservation as also pointed out by Taylor et al. (2005).

Only 5% of the people were cultivating one or two medicinal plants in small scale in the backyard. These species were *P*. *polyphylla, L*. *neesiana*, *B*. ciliata and *S*. *chirayita*. Some of the species fall under different conservation status and threat categories of different conservation agencies. For example, *Euphorbia royleana* Boiss [Euphorbiaceae], *Dioscorea deltoidea* Wall. ex Griseb. [Dioscoreaceae], *Alsophila spinulosa* (Wall. ex Hook.) R. M. Tryon [Cytheaceae], *Nardostachys jatamansi* C.B Clarke. [Caprifoliaceae], *Taxus wallichiana* Zucc. [Taxaceae] *D*. *amoenum* and *Coelogyne cristata* Lindl. [Orchidaceae]. are listed in Convention on International Trade in Endangered Species of Wild Fauna and Flora (CITES) Appendix II category. Also, some species included in the International Union for Conservation of Nature (IUCN) red list category are *N*. *jatamansi*. (Critically Endangered), *T. wallichiana* Zucc. (Endangered) and *P*. *polyphylla* (Vulnerable). Twenty-two species also fall under Conservation Assessment Management Plan (CAMP). Species under CAMP include *Aconitum spicatum* (Brühl) Stapf [Ranunuculaceae], *A*. *racemosus*, *Maharanga emodi* (Wall.) A.DC. [Boraginaceae] *etc.* This reveals that these species need more attention for conservation than other species. As there is high diversity of medicinal plants, the conservation of medicinal plants also means conservation of plant biodiversity (Hamilton, 2004). Therefore, the actions and plans should be formulated accordingly.

## Conclusion

This is a comprehensive ethnobotanical study which evaluates traditional medicinal plants used by the Gurung community in Sikles region in western Nepal. A total of 115 medicinal plant species were investigated and recorded, reflecting the rich traditional knowledge about medicinal plants, that plays an important role in local healthcare. *N*. *scrophulariiflora*, *P*. *polyphylla, L*. neesiana¸ *D*. *hatagirea* and *B*. *ciliata* were frequently used species in terms of use values. Gurung people had consistency on the treatment of diseases in the digestive system, cough, cold and external injuries like cut and wounds. In this study, we reported two species which have not been previously recorded as medicine and also reported high number of medicinal plants. In addition, with these ethnobotanical studies, traditional knowledge will be preserved and a basic preliminary resource will be provided for further specialized studies of phytochemistry and pharmacological research for the discovery of new drugs. Therefore, further studies on such chemical composition and pharmacological activity are needed on those species which have high use values. Nowadays, traditional knowledge has seriously been threatened due to recent human activities and environmental degradation. In the future, different new approaches will be used to demystify traditional medicine. Measures are urgently needed to promote the inheritance of traditional knowledge. Also, the sustainable use of the medicinal plants needs to be ensured to improve the economic development of the local people based on the premise of biodiversity conservation.

## Data Availability

The original contributions presented in the study are included in the article/Supplementary Material, further inquiries can be directed to the corresponding authors.
