# Supplementary material for: Diversity, distribution, and sustainability of traditional medicinal plants in Kaski district, western Nepal
Source: Front Pharmacol. 2022 Dec 20;13:1076351. doi: 10.3389/fphar.2022.1076351 (PMC9807671; doi:10.3389/fphar.2022.1076351)
Supplement: Supplementary file 1 [file DataSheet1.docx]

**Supplementary materials for**

Diversity, Distribution, and Sustainability of Traditional Medicinal Plants in Kaski District, Western Nepal

Dhruba Khakurel, Yadav Uprety, Gyeongik Ahn, Joon-Yung Cha, Woe-Yeon Kim, Sung-Ho Lee, Sangeeta Rajbhandary

Correspondence to Yadav Uprety, Woe-Yeon Kim, and Sung-Ho Lee

Emails: yadavuprety@gmail.com, [kim1312@gnu.ac.kr](mailto:kim1312@gnu.ac.kr), & [leesh@gnu.ac.kr](mailto:leesh@gnu.ac.kr)

Supplementary materials contain three Supplementary tables.

| **S.N.**  Supplementary Table 1: List of ethnomedicinal plants from study area. | **Family** | **Scientific Name** | **English Name** | **Nepali Name** | **Gurung Name** | **Relative Frequency of citation (RFC)** | **Use Value (UV)** | **Life Form** | **Parts Used** | **Parts used and traditional uses** | **Collection number** | **Conservation status** |
| --- | --- | --- | --- | --- | --- | --- | --- | --- | --- | --- | --- | --- |
| **Fern and Fern allies** |  |  |  |  |  |  |  |  |  |  |  |  |
| 1 | Cytheaceae | *Alsophila spinulosa* (Wall. ex Hook.) R. M. Tryon | Tree Fern | Chatre uniyu | Chatre | 0.17 | 1.33 | T | Ys | Young shoots are used as vegetables for strength and vitality. Inner stem pith is eaten in fever. | KSD618 | CITES-Appendix II |
| 2 | Equisetaceae | *Equisetum arvense* L. | Common Horsetail | Ankle jhar | Simyu Thu | 0.14 | 1.00 | H | Wp | Plant paste is used to stop bleeding from cut and wounds. | KSD615 |  |
| 3 | Lycopodiaceae | *Lycopodium clavatum* L. | Club Moss | Nagbeli | Chhyoumai | 0.19 | 1.38 | C | Wp | Plant paste is used in skin diseases. | KSD558 |  |
| 4 | Schizaeaceae | *Lygodium japonicum* (Thunb.) Sw. | Japanese Climbing Fern | Janai lahara |  | 0.11 | 1.00 | C | Wp | Stem paste is used in mumps and small wounds in the body. | KSD625 |  |
| 5 | Polypodiaceae | *Nephrolepis cordifolia* (L.) Presl | Sword Fern | Pani amala | Kyulfu | 0.14 | 1.00 | H | Tu | Tuber of plant after cleaning is directly eaten during fever. | KSD625 |  |
| 6 | Pteridaceae | *Hemionitis albomarginata*  (C.B. Cl.) Ching |  | Ranisinka | Singi | 0.20 | 1.00 | H | Wp | Leaf paste is applied externally in cuts and wounds. | KSD528 |  |
| 7 |  | *Aleuritopteris rufa* (D. Don) Ching |  | Ranisinka | Singi | 0.14 | 1.80 | H | Wp | Juice of whole plant is eaten during fever and dysentery. | KSD565 |  |
| 8 | Selaginellaceae | *Selaginella pennata* (D. Don) Spring |  |  | Chyoni | 0.11 | 1.00 | H | Wp | Leaf paste is used in wounds. | KSD628 |  |
| 9 | Tectariaceae | *Tectaria coadunata* (J. Sm.) C. Chr. |  | Kalo Niguro | Niguro | 0.24 | 1.82 | H | Ys | Cooked Vegetables of young shoot controls stomachache and indigestion. | KSD635 |  |
| **Gymnosperms** |  |  |  |  |  |  |  |  |  |  |  |  |
| 1 | Cupressaceae | *Juniperus squamata* Buch.-Ham. ex D. Don | Flaky Juniper | Dhupi | Siuri Dhup | 0.26 | 1.00 | T | Lf | Dried leaves of the plant is mixed with dried whole plant powder of *Hymenidium benthamii*. The mixture is burned and the smoke is inhaled to cure cough and cold. | KSD29 |  |
| 2 | Taxaceae | *Taxus wallichiana* Zucc. | Himalayan Yew | Lothe salla | Saij/ Sangi | 0.11 | 1.00 | T | Ba | Bark paste is used in the skin cracks (*Goda futeko*). | KSD18 | IUCN- Endangered  CITES-Appendix II |
| **Angiosperms** |  |  |  |  |  |  |  |  |  |  |  |  |
| 1 | Acoraceae | *Acorus calamus* L. | Sweet Flag | Bojho | Chhyodomai | 0.27 | 2.21 | H | Rh | Small piece of fresh or dried rhizome is chewed for cough or sore throat. Rhizome paste is used to treat scabies. Used as insecticides. | KSD368 |  |
| 2 | Actinidiaceae | *Saurauia napaulensis* DC. |  | Gogan | Odoo | 0.16 | 1.18 | T | Fr | Ripe fruit is eaten to cure cough and cold. | KSD19 |  |
| 3 | Amaranthaceae | *Achyranthes aspera* L. | Prickly Chaff Flower | Datiwan | Datiwan | 0.17 | 1.50 | H | Ro | Root juice is given in fever and typhoid. | KSD391 |  |
| 4 |  | *Chenopodium album* L. | White Goosefoot | Bethe | Lainu | 0.24 | 1.53 | H | Se | Seeds are eaten to treat stomachache. |  |  |
| 5 | Amaryllidaceae | *Allium wallichii* Kunth | Himalayan Onion | Ban Lashun | Ban Nhoo | 0.27 | 1.11 | H | Ys | Vegetable prepared from leaves is taken as a tonic. . | KSD97 |  |
| 6 | Anacardiaceae | *Choerospondias axillaris* (Roxb.) B.L.Burtt & A.W. Hill | Nepali Hog plum | Lapsi | Kalah | 0.16 | 1.18 | T | Ba | Bark paste is used in the fracture and joint pain. | KSD357 |  |
| 7 | Apiaceae | *Centella asiatica* (L.) Urb. | Indian Pennywort | Ghodtapre | Nho mai | 0.20 | 1.43 | H | Wp | Whole plant juice is eaten to cure headache. Leaf paste is used to cure cuts. | KSD43 |  |
| 8 |  | *Hymenidium benthamii* (Wall. ex DC.) M.G. Pimenov & E.v. Kljuykov |  | Gadano | Dhanesh ful | 0.26 | 1.28 | H | Wp | The dried leaves of plant mixed with dried leaves powder of *Juniperus squamata*. The mixture is burned and get the smoked to cure cough and cold. | KSD28 |  |
| 9 |  | *Ligusticopsis wallichiana* (DC.) Pimenov & Kljuykov | Milk Parsely | Bhutkesh |  | 0.23 | 1.19 | H | Ro | Decoction of root is used to cure rheumatism. Root paste is used in body pain. | KSD587 |  |
| 10 | Araceae | *Arisaema costatum* (Wall.) Mart. |  | Sarpa Makai | Tobyo | 0.14 | 1.20 | H | Tu | Fresh tubers are considered as poisonous but used after detoxification. | KSD38 |  |
| 11 |  | *Arisaema nepenthoides* (Wall.) Mart. | Pitcher Cobra Lily | Kag Makai |  | 0.13 | 1.00 | H | Tu | The plant is poisonous. | KSD28 |  |
| 12 | Asparagaceae | *Agave americana* L. | Century Plant | Ketuki |  | 0.20 | 1.00 | S | Lf | Leaf paste is applied in external pain like gout. | KSD22 |  |
| 13 |  | *Asparagus racemosus* Willd. | Asparagus | Kurilo/Satawari | Puchu Touru | 0.43 | 1.53 | S | Ro, Ys | Young shoot are used as vegetables which gives strength to body. Root is used to increase lactation. | KSD20 |  |
| 14 |  | *Polygonatum cirrhifolium* (Wall.) Royle | Coiling Leaf Polygonatum | Khrimula | Khirimla | 0.20 | 1.00 | H | Ys | Cooked shoots taken as tonic. | KSD21 |  |
| 15 | Asteraceae | *Ageratina adenophora** (Spreng.) R. King & H. Rob. | Catweed | Banmara | Kalo Jhar | 0.24 | 1.00 | H | Lf | Paste of the leaves is applied to stop bleeding from cut and wounds. | KSD20 |  |
| 16 |  | *Ageratum conyzoides* * L. | Goat Weed | Gane | Thanau | 0.26 | 1.00 | H | Lf | Paste of the leaves is applied to stop bleeding from cut. | KSD52 |  |
| 17 |  | *Anaphalis contorta* (D. Don) Hook. f. | Eared Leaf Pearly Everlasting | Buki Ful | Tobu | 0.16 | 1.00 | H | Lf | Leaf is rubbed around pained parts to relief from pain. | KSD714 |  |
| 18 |  | *Artemisia indica* Willd. | Indian Wormwort | Titepati | Chewuri | 0.30 | 1.71 | H | Wp | Leaf juice is applied in the wounds. Leaf is used to control stomach pain. Used as insecticides. | KSD05 |  |
| 19 |  | *Cirsium verutum* (D. Don) Spreng. | Common Thistle | Thakailo | Popuche | 0.20 | 1.21 | H | Ro | Root juice is taken to control high fever. Inner part of stem is eaten fresh in fever. | KSD44 |  |
| 20 |  | *Taraxacum officinale* Weber ex Wiggins | Dandelions | Tuki Phool |  | 0.24 | 1.00 | H | Lf | Leaf paste is applied in cut and wounds. | KSD63 |  |
| 21 | Begoniaceae | *Begonia picta* Sm. | Painted Leaf Begonia | Magar Kanche | Prugyu | 0.23 | 1.00 | H | Ro, St | Root and stem are chewed to cure constipation. | KSD98 |  |
| 22 | Berberidaceae | *Berberis aristata* DC. | Indian Barberry | Chutro | Tishya | 0.20 | 1.00 | S | Ro | Extract from root bark is given to cure jaundice (*Pahele*). | KSD06 |  |
| 23 |  | *Berberis concinna* Hook. fil. |  | Chutro |  | 0.26 | 1.06 | S | Ba, Ro | Root juice is given to cure fever. | KSD791 |  |
| 24 |  | *Berberis napaulensis* DC. | Nepal Barberry | Thulo chutro |  | 0.16 | 1.73 | S | Br, Fr | Bark juice is taken during dysentery. Fruit is helpful in urinary infections | KSD23 |  |
| 25 | Boraginaceae | *Cynoglossum zeylanicum** (Vahl) Thub. ex Lehm. |  | Kanike kuro | Khil tana | 0.11 | 1.00 | H | Lf | Leaf paste is used in cuts. | KSD45 |  |
| 26 |  | *Maharanga emodi* (Wall.) A.DC. |  | Maharangi |  | 0.11 | 1.75 | H | Ro | Root paste is used to control worm in stomach and also used in eye problems. | KSD107 |  |
| 27 | Campanulaceae | *Lobelia pyramidalis* Wall. |  | Eklebir |  | 0.13 | 1.22 | H | Lf, Fl | Root and stem are chewed to cure constipation. | KSD46 |  |
| 28 | Cannabaceae | *Cannabis sativa* * L. | Hemb | Ganja | Vamm | 0.20 | 1.36 | H | Se | Seeds are eaten to cure stomachache. | KSD47 |  |
| 29 | Caprifoliaceae | *Dipsacus inermis* Wall. | Himalayan Teasel | Ban Karyal |  | 0.13 | 1.00 | H | Lf | Leaf paste is used in the cut and wounds. | KSD786 |  |
| 30 |  | *Nardostachys jatamansi* C..B Clarke. |  | Bhutle | Jaramansi | 0.21 | 1.13 | H | Rh | Rhizome is used in mensuration problems (eaten during pain). | KSD633 | IUCN- Critically Endangered  CITES-Appendix II |
| 31 | Caryophyllaceae | *Drymaria cordata* (Blume) J.A. Duke | Tropical Cheekweed | Abi jalo | Sya Nho | 0.24 | 1.53 | H | Wp | Plant is crushed and inhaled to treat sinusitis; plant paste is also applied on forehead to treat headache. | KSD792 |  |
| 32 | Convolvulaceae | *Cuscuta reflexa* Roxb. | Giant Dodder | Aakasa beli |  | 0.20 | 1.57 | C | Wp | Whole plant juice is taken to cure high fever and jaundice. | KSD156 |  |
| 33 | Coriariaceae | *Coriaria nepalensis* Wall. | Masuri Berry | Machaino |  | 0.13 | 1.00 | S | Lf | Leaves are poisonous. | KSD281 |  |
| 34 | Cucurbitaceae | *Solena amplexicaulis* (Lam.) Gandhi | Creeping Cucumber | Gol kakro | Toju | 0.26 | 1.44 | C | Ro | Juice obtained from the root is used to treat high fever and stomachache. | KSD48 |  |
| 35 |  | *Trichosanthes tricuspidata* Lour. | Indrayan | Indreni lahara |  | 0.11 | 1.00 | C | Se | Roasted seeds are eaten to control vomiting. | KSD776 |  |
| 36 | Dioscoreaceae | *Dioscorea deltoidea* Wall. ex Griseb. | Nepal Yam | Ban tarul | Teeme | 0.20 | 1.00 | C | Tu | Tuber is cooked and eaten during high fever. | KSD403 |  |
| 37 | Elaeagnaceae | *Elaeagnus parvifolia* Wall. | Autumn Olive | Guheli | Tuwuru | 0.16 | 1.55 | T | Ba, Fr | Bark paste is used in the fracture. Fruit is eaten during stomach problems. | KSD11 |  |
| 38 | Ericaceae | *Lyonia ovalifolia* (Wall.) Drude | Oval Leaf Lyonia | Angeri | Chayarsi | 0.30 | 1.52 | T | Ro, Lf | Root paste is used in skin diseases. Young leaves are poisonous. | KSD13 |  |
| 39 |  | *Rhododendron arboreum* Sm. | Tree Rhododendron | Laligurans | Pori | 0.23 | 1.69 | T | Fl, Ba | Flower paste is chewed in case of fish bone stuck in the throat and also for stomach ache, and dysentery. | KSD100 |  |
| 40 | Euphorbiaceae | *Euphorbia royleana* Boiss. | Royal's Spurge | Siudi |  | 0.11 | 1.50 | S | Lt | Latex of plant is considered good for muscular swellings. Inner part of the stem (pith) is eaten during stomach pain and menstrual disorders. | KSD652 |  |
| 41 | Fabaceae | *Erythrina arborescens* Roxb. | Himalayan Coral Tree | Tekhi katha(Faledo) |  | 0.14 | 1.10 | T | Ba | Bark juice is eaten during dysentery. | KSD761 |  |
| 42 | Fagaceae | *Quercus lanata* Sm. | Wolly Leaved Oak | Bajha |  | 0.16 | 1.18 | T | Ba | Bark paste is used in muscular problems and body pain. | KSD735 |  |
| 43 | Gentianaceae | *Swertia angustifolia* Buch.-Ham. ex D. Don | Narrow Leaved Swertia | Chiraita | Titte | 0.34 | 1.38 | H | Wp | Infusion of the plant is taken in high fever. | KSD51 |  |
| 44 |  | *Swertia chirayita* (Roxb.) H. Karst. | Chirayita | Chiraita | Titte | 0.84 | 3.50 | H | Wp | Decoction of the plant is used in reducing the fever and stomach problems. | KSD50 |  |
| 45 |  | *Swertia paniculata* Wall. | Panicled Swertia | Lekh Chiraita | Lekh Title | 0.23 | 1.69 | H | Wp | Whole plant is used in fever and cold. | KSD78 |  |
| 46 |  | *Halenia elliptica* D. Don | Spurred Gentian | Titte |  | 0.17 | 1.42 | H | Wp | Roots are boiled and taken during high fever. | KSD70 |  |
| 47 | Juglandaceae | *Juglans regia* L. | Walnut | Okhar |  | 0.20 | 1.00 | T | Lf, Ba | Bark and leaf paste is used in skin diseases. | KSD623 |  |
| 48 |  | *Engelhardia spicata* Lechen ex Bl. | Mauwa | Mouwa | Krow | 0.11 | 1.00 | T | Lf | Leaves are poisonous. | KSD731 |  |
| 49 | Lamiaceae | *Colebrookea oppositifolia* Sm. | Indian Squirrel Tail | Dhurselo |  | 0.11 | 1.00 | S | Ro | Root juice is given to cure high fever. | KSD36 |  |
| 50 |  | *Elsholtzia blanda* (Benth.) Benth. | Pleasant Himalayan Mint | Ban Silam |  | 0.11 | 1.00 | H | Lf | Leaf paste is used as leech repellent. | KSD66 |  |
| 51 |  | *Leucosceptrum canum* Sm. | Hairy White - Wand | Ghurmiso | Cheena Dhu | 0.26 | 1.22 | T | Ro | Root paste is applied in wounds. | KSD32 |  |
| 52 | Lauraceae | *Cinnamomum tamala* (Buch.-Ham.) Th. G. G. Nees | Indian Bay Leaf | Tejpat | Lepu | 0.36 | 1.68 | T | Ba, Lf | Bark paste is used to control the fever. Leaf is used to cure cough and cold. | KSD55 |  |
| 53 |  | *Lindera neesiana* (Wall. ex Nees) Kruz |  | Siltimur | Kudu | 0.69 | 3.05 | T | Fr | Fruits are eaten in gastritis, stomach swelling and vomiting. Dried fruit powder is used in tea as stimulant. | KSD33 |  |
| 54 | Liliaceae | *Fritillaria cirrhosa* D. Don | Himalayan Fritillary | Ban Lasun |  | 0.24 | 1.35 | H | Tu | Tuber is eaten to control stomach disorders and cough. | KSD56 |  |
| 55 | Linaceae | *Reinwardtia indica* Dumort. | Yellow Flax | Pyauli |  | 0.17 | 1.50 | S | Lf | Leaf paste is used in small mumps and scorpion bites. | KSD788 |  |
| 56 | Malvaceae | *Bombax ceiba* L. | Silk Cotton Tree | Simal |  | 0.27 | 1.21 | T | Ro | Root paste is used to cure cut and wounds. | KSD763 |  |
| 57 | Melanthiaceae | *Paris polyphylla* Sm. | Himalayan Paris | Satuwa | Sadwaa | 0.77 | 3.70 | H | Rh | Rhizome is applied in cut, wounds and burns. The rhizome chewed or decoction is eaten to prevent stomachache. | KSD57 | IUCN- Vulnerable |
| 58 | Moraceae | *Ficus auriculata* Lour. | Elephant Ear Fig | Nimaro | Toubu | 0.17 | 1.08 | T | Lt | Latex is used in cut and wounds. | KSD87 |  |
| 59 | Myricaceae | *Myrica esculenta* (Buch.-Ham. ex D. Don) I.M. Turner | Box Myrtle | Kafal |  | 0.20 | 1.64 | T | Ba | Bark decoction is used to cure diarrhea, dysentery and chronic bronchitis. | KSD714 |  |
| 60 | Orchidaceae | *Dactylorhiza hatagirea* (D. Don) Soó | Himalayan Marsh Orchid | Panchaule | Yori-nghee | 0.63 | 2.13 | H | Ro | Piece of rhizome is chewed to cure stomachache and headache. Paste of rhizome is used in burn. | KSD58 | CITES-Appendix II |
| 61 |  | *Dendrobium amoenum* Wall.ex Lindl. | Lovely Dendrobium | Hadjorne | Hadjor | 0.09 | 1.00 | H | Pb | Pseudo bulb are crushed and paste mixed with *Rheum australe* is used in the fracture. | KSD96 | CITES-Appendix II |
| 62 |  | *Coelogyne cristata* Lindl.. | Crested Coelogyne | Chadigava | Salleta | 0.11 | 1.00 | H | Pb | Pseudo bulb of plants is used for small wounds. | KSD77 | CITES-Appendix II |
| 63 | Orobanchaceae | *Pedicularis siphonantha* D. Don | Tube Lousewort |  | Herbeta | 0.26 | 1.28 | H | Ro | Root juice is used to control the stomach pain. | KSD59 |  |
| 64 | Oxalidaceae | *Oxalis corniculata* L. | Creeping Wood Sorrel | Chariamilo |  | 0.23 | 1.31 | H | Lf | Infusion of leaves is used to cure eye problems. | KSD17 |  |
| 65 | Phyllanthaceae | *Phyllanthus parvifolius* Buch-Ham. ex D. Don |  | Khareto |  | 0.20 | 1.14 | H | Lf | Paste of leaves is used in muscles pain. | KSD08 |  |
| 66 | Phytolaccaceae | *Phytolacca acinosa* Roxb. | Indian Pokeweed | Jaringo sag | Olitaha | 0.14 | 1.80 | H | Ys | Cooked vegetables of young shoot is used to control stomachache and diarrhea | KSD60 |  |
| 67 | Piperaceae | *Piper mullesua* Buch.-Ham. ex D. Don | Hill Pepper | Pahadi pan | Sindri | 0.19 | 1.92 | H | Fr | Fruits are used to cure respiratory problems and cough. | KSD42 |  |
| 68 | Plantaginaceae | *Neopicrorhiza scrophulariiflora* (Pennell) D.Y. Hong |  | Kutki | Kutki | 0.80 | 2.18 | H | Rh | Roots are used for high fever, intestinal problems and stomachache. | KSD61 |  |
| 69 |  | *Plantago major* L. | Broad Leaf Plantain |  |  | 0.20 | 1.32 | H | Wp | Seeds are chewed and swallowed to treat dysentery Infusion of the plant is drunk to treat urinary disorders. | KSD701 |  |
| 70 | Poaceae | *Cynodon dactylon* (L.) Pers. | Bermuda Grass | Dubo | Tu Nho | 0.27 | 1.32 | H | Wp | Plant juice is eaten during urinary problems. | KSD15 |  |
| 71 | Polygonaceae | *Rheum acuminatum* Hook. fil. & Thoms. | Ornamental Rhubarb | Padamchal | Thulo Pudumchalne | 0.41 | 1.21 | H | Wp | Plant is used as substitute of *Rheum australe* and used in fracture. | KSD771 |  |
| 72 |  | *Rheum australe* D. Don | Himalayan Rubarb | Padamchal | Pudumchalne | 0.50 | 2.84 | H | Wp | Root paste is applied in sprain and fractures. It is also taken to relief from headache. Juice of the shoot portion is taken in dysentery and intestinal problems. Dried root powder is used as tea for body ache. | KSD166 |  |
| 73 |  | *Rumex nepalensis* Spreng. | Nepal Dock | Halhale | Olmi | 0.37 | 1.12 | H | Ro | Cleaned roots are crushed with water and the paste is applied externally to cure wounds. | KSD62 |  |
| 74 | Primulaceae | *Maesa chisia* Buch.-Ham. ex D. Don |  | Bilaune | Chode | 0.23 | 1.13 | S | Fr | Fruits are used to cure skin problems. | KSD344 |  |
| 75 | Ranunculaceae | *Aconitum gammiei* Stapf |  | Nirmashi | Naramashi | 0.40 | 1.36 | H | Tu | Tuber are used for stomach ache and fever. | KSD366 |  |
| 76 |  | *Aconitum spicatum* (Brühl) Stapf | Nepali Aconite | Bikha | Megai | 0.27 | 1.37 | H | Tu | Poisonous | KSD365 |  |
| 77 |  | *Delphinium vestitum* Wall. ex Royle | Hairy Delphinium | Mawarmul | Narmashi | 0.23 | 1.19 | H | Ro | Roots are used as anthelminthic. | KSD120 |  |
| 78 |  | *Thalictrum foliolosum* DC. | Leafy Meadow Rue | Dampate | Kaude | 0.24 | 1.53 | H | Ro | Root juice is given to cure diarrhea and fever. | KSD119 |  |
| 79 | Rosaceae | *Argentina lineata* (Trevir.) Sojak | Lined Cinquefoil | Bajradanti | Hosre | 0.21 | 1.80 | H | Ro | Cleaned root is chewed to treat toothache. Fresh roots were taken during the abdominal pains. | KSD79 |  |
| 80 |  | *Fragaria nubicola* Lindl. | Himalayan Strawberry | Bhui kafal |  | 0.23 | 1.14 | H | Ro, Fr | Root paste is used in controlling bleeding, cough and cold. Fruit is taken as digestive and laxative. | KSD344 |  |
| 81 |  | *Prunus cerasoides* D. Don | Wild Himalayan Cherry | Painyu | Chyarbu | 0.20 | 1.14 | T | Lf, Ba | Leaves and bark paste is used in fractures. | KSD16 |  |
| 82 |  | *Pyracantha crenulata* (Roxb. ex D. Don) M. Roemer | Nepalese Firethorn | Ghangaru |  | 0.16 | 1.55 | S | Fr | Ripe fruits are eaten to cure diarrhea and dysentery. | KSD26 |  |
| 83 |  | *Rubus ellipticus* Sm. | Yellow Himalayan Raspberry | Aiselo | Palah | 0.40 | 1.64 | S | Ro | Root juice is used to cure typhoid and stomach disorders. | KSD87 |  |
| 84 |  | *Prinsepia utilis* Royle | Himalayan Cherry Prinsepia | Dhatelo | Baimai tisya | 0.19 | 1.23 | S | Se | Oil is extracted from the seeds and used to relief from pain. | KSD37 |  |
| 85 | Rubiaceae | *Galium elegans* Wall. ex Roxb. | Elegant Goose Grass | Lahare kuro |  | 0.13 | 1.78 | C | Lf | Leaf paste is used to control bleeding from cuts. | KSD110 |  |
| 86 |  | *Rubia manjith* Roxb. | Manjith | Machitoo | Lera | 0.11 | 1.78 | C | Ro | Root paste is used to cure skin diseases and scorpion bites. | KSD09 |  |
| 87 | Rutaceae | *Zanthoxylum armatum* DC. | Winged prickly Ash | Timur | Purmo | 0.74 | 1.58 | T | Fr | Dried fruit powder is used to cure stomach pain and indigestion. | KSD311 |  |
| 88 |  | *Boenninghausenia albiflora* (Hook.) Rchb. ex Meisn. | White Himalayan Rue | Ankuree jhar | Damapate | 0.10 | 1.00 | H | Wp | Insecticide. | KSD123 |  |
| 89 | Santalaceae | *Pyrularia edulis* (Wall.) A. DC. |  | Amphi | Amphi | 0.13 | 1.00 | T | Se | Oil extracted from the seeds is used in skin cracks (*Goda futeko*). | KSD317 |  |
| 90 | Saururaceae | *Houttuynia cordata* Thunb. | Chameleon Plant | Gandhe | Fitta dhu | 0.07 | 1.60 | H | Lf | Leaf paste is used in cut and wounds. | KSD122 |  |
| 91 | Saxifragaceae | *Astilbe rivularis* Buch.-Ham. ex D. Don | River Astilbe | Thulo Okhati | Okhata | 0.39 | 2.22 | S | Ro | Dried root powder is given to post-natal women to cure the body of women. Also used in diarrhea and dysentery. | KSD121 |  |
| 92 |  | *Bergenia ciliata* (Haw.) Sternb. | Hairy Bergenia | Pakhan bed | Ghuru basana | 0.56 | 2.54 | H | Rh | Dried root powder is given to post-natal women to cure the body of women. Root powder is also used in diarrhea and dysentery. | KSD324 |  |
| 93 | Scrophulariaceae | *Buddleja paniculata* Lour. | Panicled Butterfly Bush | Bhimsenpati |  | 0.14 | 1.00 | T | Lf, Fl | Leaf is used in fermentation and flower is used for fish poisoning. | KSD308 |  |
| 94 | Simaroubaceae | *Brucea javanica* (L.) Merr. | Brucea | Bhakiamilo | Tiuwru | 0.29 | 1.75 | T | Fr | Powder of fruits is used to control diarrhea and dysentery. | KSD305 |  |
| 95 | Smilacaceae | *Smilax aspera* L. | Common Smilax | Kukurdaino | Neridhu | 0.11 | 1.38 | C | Ro | Root paste is used to cure skin problems. | KSD27 |  |
| 96 | Solanaceae | *Nicotiana tabacum* L. | Tobacco | Kacho pat | Tama po | 0.06 | 0.66 | S | Lf | Leaf paste is use to cure ear problems. | KSD10 |  |
| 97 |  | *Solanum nigrum* L. | Black Nightshade | Kali gedi | Taujarmai | 0.10 | 1.29 | H | Lf | Leaf paste is applied in cut and wounds. | KSD307 |  |
| 98 |  | *Solanum aculeatissimum* Jacq. | Thorny Nightshade | Kantakari | Sa Naba Mai | 0.11 | 1.00 | H | Se | Seeds are used to treat toothache | KSD304 |  |
| 99 | Theaceae | *Schima wallichii* Choisy | Schima | Chilaune | Jhoose | 0.14 | 1.00 | T | Ba | Paste of bark is applied in the bone fracture. | KSD801 |  |
| 100 | Urticaceae | *Girardinia diversifolia* (Link) Friis | Giant Asian Nettles | Allo | Ni polu | 0.16 | 1.45 | H | Ro | Root juice is used in the stomach problems. | KSD01 |  |
| 101 |  | *Urtica dioica* L. | Stinging Nettle | Sisnu | Polu | 0.37 | 1.12 | H | Wp | Root juice is used to cure fever. Young shoot is eaten as vegetables to reduce joint pain. | KSD02 |  |
| 102 | Violaceae | *Viola pilosa* Bl. | Smooth-Leaf White Violet | Ghatte pul | Bou mai | 0.21 | 1.20 | H | Wp | Whole plant paste is used to cure wounds. | KSD64 |  |
| 103 | Vitaceae | *Tetrastigma serrulatum* (Roxb.) Planch. | Toothed Leaf Chestnut Vine | Charchare |  | 0.14 | 1.00 | C | Ys | Latex from young shoot is used to cure eye infection. | KSD63 |  |
| 104 | Zingiberaceae | *Curcuma angustifolia* Roxb. | East Indian Arrowot | Barkhe Sarro |  | 0.17 | 1.00 | H | Ro | Root paste is applied in fractured and dislocated bones. | KSD153 |  |

Life Form: H- Herbs, C-Climbers, S- Shrubs, T-Trees

Parts Used: Rh-Rhizome, Ro-Root, Wp-Whole plant, Lf-Leaf, Ba-Bark, Ys-Young shoot, Fr- Fruit, Fl- Flower, Lt- Latex, Se- Seed, St- Stem, Tu- Tuber

Conservation Status: IUCN-International Union for Nature Conservation, CITES- Convention on International Trade in Endangered Species of Wild Fauna and Flora

* Exotic Species

Supplementary Table 2: Summary stats for Relative Frequency of Citation (RFC) and Use Value (UV)

|  | Mean | Standard Deviation | Minimum | Maximum |
| --- | --- | --- | --- | --- |
| RFC | 0.23 | 0.19 | 0.06 | 0.84 |
| UV | 1.38 | 0.45 | 0.66 | 3.7 |

Supplementary Table 3: Association between RFC and UV using Pearson correlation method

| Correlation coefficient (r) | 0.63 |
| --- | --- |
| Coefficient of determination (r2) | 0.4 |
| p Value | 0.00006 |
